# Extraction and Quantification of Sulforaphane and Indole-3-Carbinol from Rapeseed Tissues Using QuEChERS Coupled with UHPLC-MS/MS

**DOI:** 10.3390/molecules25092149

**Published:** 2020-05-04

**Authors:** Xu Yu, Fei Ma, Liangxiao Zhang, Peiwu Li

**Affiliations:** 1 Oil Crops Research Institute, Chinese Academy of Agricultural Sciences, Wuhan 430062, China; yuxu@caas.cn (X.Y.); zhanglx@caas.cn (L.Z.); peiwuli@oilcrops.cn (P.L.); 2 Key Laboratory of Biology and Genetic Improvement of Oil Crops, Ministry of Agriculture and Rural Affairs, Wuhan 430062, China; 3 Key Laboratory of Detection for Mycotoxins, Ministry of Agriculture and Rural Affairs, Wuhan 430062, China; 4 Laboratory of Quality and Safety Risk Assessment for Oilseeds Products (Wuhan), Ministry of Agriculture and Rural Affairs, Wuhan 430062, China; 5 Quality Inspection and Test Center for Oilseeds Products, Ministry of Agriculture and Rural Affairs, Wuhan 430062, China

**Keywords:** sulforaphane, indole-3-carbinol, QuEChERS, quantification, UHPLC-MS/MS

## Abstract

Rapeseed (*Brassica napus L*.) is rich in phenols, vitamins, carotenoids, and mineral elements, such as selenium. Additionally, it contains the active ingredients sulforaphane and indole-3-carbinol, which have been demonstrated to have pharmacological effects. In this study, sulforaphane and indole-3-carbinol were extracted and quantified from rapeseeds using quick, easy, cheap, effective, rugged and safe (QuEChERS) method coupled with ultra high performance liquid chromarography tandem mass spectrometry (UHPLC-MS/MS). The major parameters for extraction and purification efficiency were optimized, including the hydrolysis reaction, extraction condition and type and amount of purification adsorbents. The limit of detection (LOD) and the limit of quantification (LOQ) for sulforaphane were 0.05 μg/kg and 0.15 μg/kg, and for indole-3-carbinol were 5 μg/kg and 15 μg/kg, respectively. The developed method was used to successfully analyze fifty rapeseed samples. The QuEChERS coupled with UHPLC-MS/MS simultaneously detect sulforaphane and indole-3-carbinol in vegetable matrix and evaluate the quality and nutrition of rapeseed samples.

## 1. Introduction

Rapeseed (*Brassica napus L*.) is a major oilseed crop in China. China generates approximately 20% of the world’s yield [1]. Rapeseeds not only produce edible oils, but their sprouts, seedlings, and leaves have nutritional value for human consumption [2]. Vegetables such as *Brassicaceae* (*Cruciferae*) are vital nutritional sources throughout the world. They contain several chemopreventive compounds, such as glucosinolates, polyphenols, and selenium [3,4,5]. During plant tissue damage, glucosinolates decompose into isothiocyanates and nitriles in the presence of the endogenous enzyme myrosinase (EC 3.2.1.147), then undergo immediate hydrolysis of the β-thioglucoside group [6].

**Figure 1:**
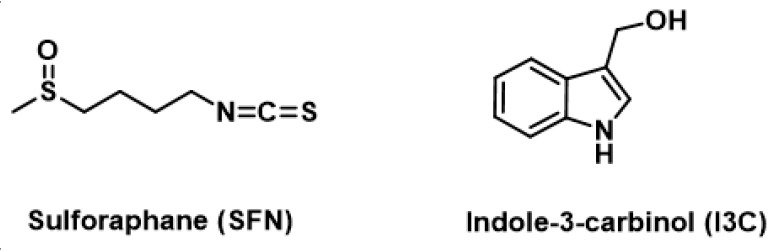
The molecular structures of sulforaphane and indole-3-carbinol.

More than 300 glucosinolates and their hydrolytic products have been identified and evaluated for their beneficial effects [7,8]. The phytochemicals classified as isothiocyanates have been widely utilized to investigate their anti-tumor, anti-cancer and anti-inflammatory effects. Sulforaphane (SFN) has been demonstrated to be the most potent compound. It is the activator of the Nrf2 pathway, which regulates the cellular defense system and promotes various detoxifying and antioxidative effects [9,10]. Sulforaphane has beneficial effects for diabetic complications by effectively reducing fasting blood glucose and glycated hemoglobin levels in obese type 2 diabetic patients with poor glycemic control [11]. The bioavailability of sulforaphane in vegetables has also been studied, and could serve as a guide for the design and implementation of SFN in clinical trials [12,13]. Indole-3-carbinol (I3C) is another important anti-cancer chemopreventive compound that acts via further hydrolysis of isothiocyanate (indol-3-methyl isothiocyanate), which is derived from cruciferous plants. The I3C compound has been shown to regulate the growth of cancer cells by modulating genes involved in growth, signal transduction and carcinogenesis [14]. It can inhibit the proliferation of cancer cells [15,16], inhibit the expression of drug resistance genes [17], and induce apoptosis [18]. Preliminary clinical trials have indicated that indole-3-carbinol could be used to protect against hormone dependent/independent human cancers [19]. As shown in Figure 1, sulforaphane and indole-3-carbinol are both non-toxic, “natural products” and are often used in combination with conventional chemotherapy to treat human malignancies to give a lower toxicity and higher efficacy.

An appropriate sample pretreatment method is a key step to the quantification of sulforaphane and indole-3-carbinol in vegetables. Current pretreatment extraction techniques for sulforaphane and indole-3-carbinol include liquid–liquid extraction [20], solid-phase extraction (SPE) [21], dispersive liquid–liquid microextraction (DLLME) [22], high-speed countercurrent chromatography (HSCCC) [23] and preparative HPLC [24]. However, the majority of the extraction and purification methods are tedious and require large volumes of organic solvents or sophisticated equipment. QuEChERS (quick, easy, cheap, effective, rugged and safe) is a comprehensive method that has been widely used to determine target analytes in foods, agri-products, environmental substances and biological fluids [25,26]. The QuEChERS method constitutes two key steps including liquid–liquid extraction and dispersive solid-phase extraction clean-up. This method is simple, versatile, eco-friendly, eliminates matrix effects and has excellent recovery. The QuEChERS is an ideal method for the extraction and purification of sulforaphane and indole-3-carbinol in vegetable samples.

Quantitative methods for sulforaphane include thin-layer chromatography (TLC) [27], gas chromatography (GC) [28], and gas chromatography-mass spectrometry (GC-MS) [29]. However, high temperatures at the injection ports in the GC or GC-MS system could result in the degradation of sulforaphane and indole-3-carbinol [30]. To measure the stability and solubility of the analytes, liquid chromatography coupled with various detectors has been developed. These include ultraviolet (UV) [31], diode-array (DAD) [32], evaporative light-scattering detectors (ELSD) [33], fluorescence detectors (FLD) [34] and high-resolution mass spectrometry (HR-MS) [35]. UHPLC-MS/MS provides high-throughput screening capability with confirmatory data. This is an important method for the simultaneous determination of nutritional compounds in plant samples.

This study aimed to develop a fast, simple and accurate method using QuEChERS coupled with UHPLC-MS/MS for the extraction and quantification of sulforaphane and indole-3-carbinol in rapeseed samples. The major parameters for extraction and purification efficiency were investigated, including the hydrolysis reaction, extraction condition and type and amount of purification adsorbents. Under the optimized parameters, the analytes of rapeseed stem tissue were simultaneously detected.

## 2. Results and Discussion

### 2.1. Optimization of Extraction Conditions 

#### 2.1.1. The Effect of Hydrolysis Time

**Figure 2:**
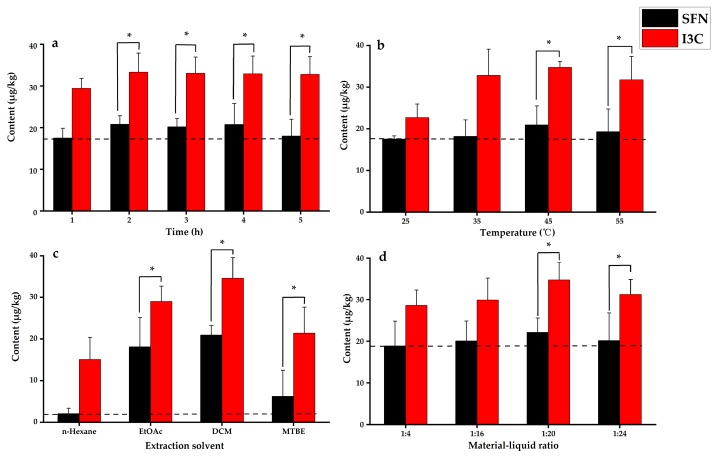
Optimized extraction conditions: (**a**) effect of hydrolysis time, (**b**) effect of temperature, (**c**) effect of solvent type, (**d**) effect of extraction solvent volume. “*” with brackets represent significant differences of SFN and I3C, *p* < 0.05.

The yield of sulforaphane and indole-3-carbinol extracted from rapeseed tissue samples before and after hydrolysis were compared. The yield of sulforaphane and indole-3-carbinol extracted without hydrolysis was 50.3%, which was 30.0% lower compared to the yield obtained after hydrolysis. The results demonstrated that the hydrolysis step increased the yield of sulforaphane and indole-3-carbinol. Enzymatic hydrolysis was performed for 1 to 5 h. An increase in sulforaphane and indole-3-carbinol yields were observed with an increase in hydrolysis times (Figure 2a). Peak yields of sulforaphane and indole-3-carbinol were observed after two hours of hydrolysis. Prolonged hydrolysis resulted in slightly lower yields of sulforaphane. Hence, the hydrolysis time was set at 2 h for subsequent experiments.

#### 2.1.2. Effect of Hydrolysis Temperature

The effect of temperature was investigated from 25 °C to 55 °C for the hydrolysis reaction. Yields for sulforaphane and indole-3-carbinol increased from 25 °C to 45 °C (Figure 2b). However, when the temperature was at 55 °C the yield for indole-3-carbinol decreased. The results indicated that endogenous myrosinase had the best catalytic activity at 45 °C. Hence, the hydrolysis temperature was set at 45 °C for subsequent experiments.

#### 2.1.3. Effect of Solvent Type on Extraction 

Different organic solvents were evaluated to improve the extraction efficiency, such as *n*-hexane, methyl tert-butyl ether, ethyl acetate, and dichloromethane [36]. As shown in Figure 2c, the high yields for sulforaphane and indole-3-carbinol were achieved with the increased polarity from 0.519 *n*-hexane to 0.876 dichloromethane. Additionally, organic and aqueous phases could be separated in short time using high density dichloromethane, which significantly improved extraction efficiency. Hence, dichloromethane was selected as the extraction solvent.

#### 2.1.4. Effect of Solvent Volume on Extraction

Appropriate extraction solvent volume could improve the complete extraction of the target compounds. Extraction yields for the two compounds at different the material–liquid ratios of 1: 4, 1:16, 1:20, and 1:24 (g/mL) are shown in Figure 2d. The extraction yields gradually increased with larger volume of extraction solvent and the extraction yields plateaued when the ratio of material to liquid reached 1:20 (g/mL). Based on factors such as reagent use, environmental pollution, test costs, and other factors, the final material–liquid ratio of 1:20 (g/mL) was selected.

### 2.2. Type and Amount of Purification Adsorbents 

**Figure 3:**
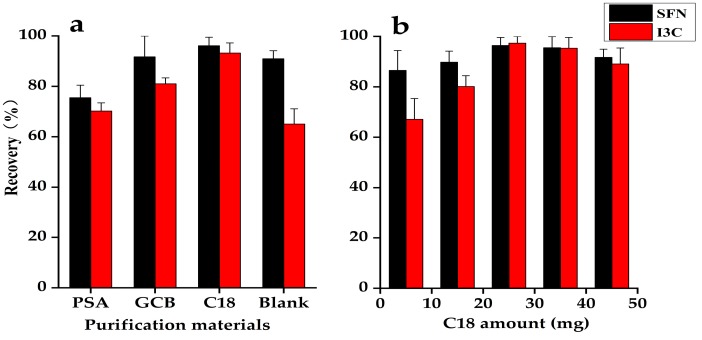
Optimization of the amount and type of purification material: (**a**) effect of the different types of purification materials, (**b**) amounts of C18 used for purification. Solutions were spiked with sulforaphane at 20 µg/kg, indole-3-carbinol at 50 µg/kg.

Different purification adsorbents including C18, PSA, GCB were evaluated for extraction steps. As shown in Figure 3a, using C18 for purification was the best, with optimal recovery for both compounds. The recoveries using PSA, GCB and no purification were low for both compounds, indicating that PSA and GCB had an adsorption effect on the target compounds. Extraction without purification had the presence of pigments and other impurities, which significantly contaminated the ion source of the mass spectrometer.

Using C18, the target compound was inhibited from ionization, resulting in a decrease in MS signal. Hence, the effects of different amounts of C18 (5, 15, 25, 35, and 45 mg) on the purification effect of the target compounds were investigated. As shown in Figure 3b, 25 mg of C18 resulted in recovery rates of 96.4% and 97.3% for sulforaphane and indole-3-carbinol, respectively, which satisfied the quantitative requirements. A decrease in recovery rate was observed when greater than 25 mg of C18 was used.

### 2.3. Method Validation

#### 2.3.1. Linear Equations, Detection Lines, and Limits of Quantification

Under optimal conditions, the peak area was designated as the ordinate and the concentration as the abscissa to establish the standard curve. The regression equation and linear range for each compound are shown in Table 1. The limit of detection (LOD) and the limit of quantification (LOQ) were determined using the target compound concentration corresponding to 3 and 10 times the signal-to-noise ratio (S/N), respectively. The LOD and LOQ for sulforaphane were 0.05 μg/kg and 0.15 μg/kg, and for indole-3-carbinol were 5 μg/kg and 15 μg/kg, respectively.

#### 2.3.2. Recovery, Intra-Day and Inter-Day Precision 

Sulforaphane and indole-3-carbinol were spiked at different concentrations in rapeseed leaf samples. The recoveries were calculated using the ratio between (total detected amount-original amount) and spiked amount [37,38]. As presented in Table 2, the recoveries of sulforaphane were in the range of 76.5%–96.4%, and indole-3-carbinol were in the range of 80.2%–97.3%. The results satisfied the acceptance criteria for the assay. Accuracy was calculated by repeated measurements and then the relative standard deviation (RSD, %) was calculated. Using the same conditions, different concentrations of the spiked matrix were measured three times within one day to assess intra-day precision. They were also measured for five consecutive days to assess inter-day precision. The intra-day and inter-day precision for sulforaphane were less than 5.9% and 11.3%, respectively, and for indole-3-carbinol were less than 10.3% and 10.6%, respectively. The results indicated that target compounds were stable.

#### 2.3.3. Matrix Effect

Components other than the analytes from rapeseed tissues were considered to be matrices. These matrices may enhance or reduce the intensity of the mass spectra. Sample spike experiments were used to determine matrix effects (ME). Three sets of experiments were designed to evaluate the matrix effects of the method. Matrix standard solution (A), matrix solution (B) and standard solution (C) with different concentrations were investigated. The matrix effect was calculated using the following formula:

ME (%) = [(A − B)/C − 1] × 100%

The ME in rapeseed tissues ranged from −6.56% to 11.02%, which indicated that this extraction and purification method was acceptable for routine UHPLC-MS/MS analysis.

### 2.4. Method Application

**Figure 4:**
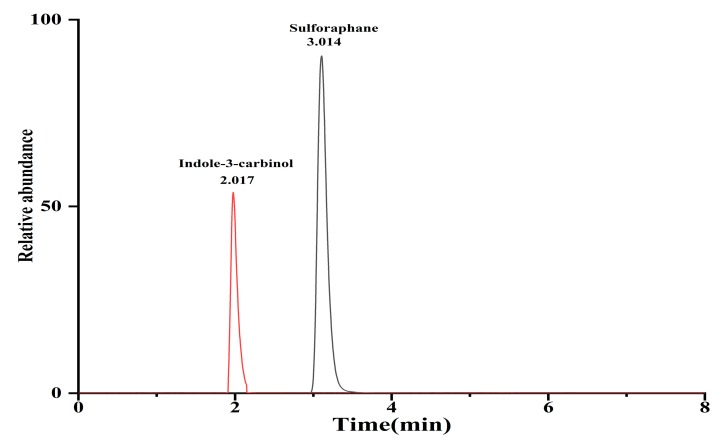
UHPLC-MS/MS chromatograms (MRM) of sulforaphane and indole-3-carbinol in rapeseed samples at 10 and 25 µg/kg, respectively.

**Figure 5:**
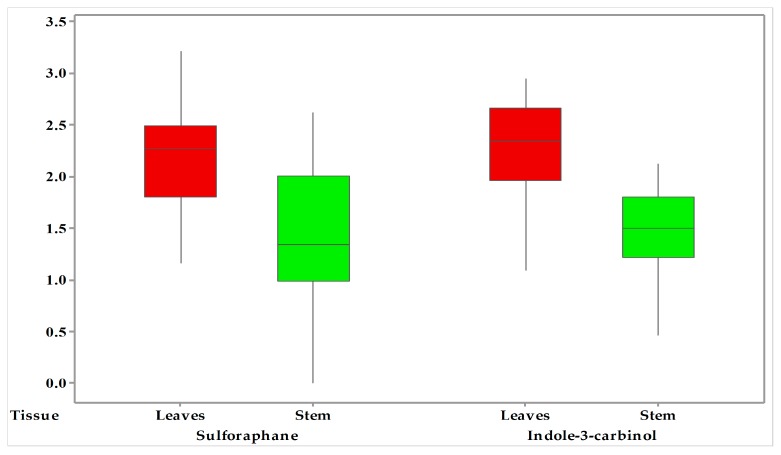
Box plot of sulforaphane and indole-3-carbinol concentration in the leaves and stems of rapeseed samples.

The developed synchronous quantitative method was applied for the determination of sulforaphane and indole-3-carbinol compounds in rapeseed tissues as present in Figure 4. The summary of sulforaphane and indole-3-carbinol in the leaves and stems derived from rapeseed are illustrated in Table 3. The overall ranges of sulforaphane and indole-3-carbinol are presented in this validation study for rapeseed tissues. The amount of sulforaphane varied from ND—415.3 μg/kg and 14.6 μg/kg—1621.8 μg/kg in stems and leaves, and the amount of indole-3-carbinol varied from ND—131.3 μg/kg and 36.4—879.5 μg/kg in stems and leaves. As depicted in Figure 5, the highest sulforaphane and indole-3-carbinol were both found in the leaf samples (*p* < 0.05). The difference in all analytes between stems and leaves could be attributed to the part, variety, geography, climate and environment conditions, in accordance with previous studies [39,40,41].

Compared to previous studies, this QuEChERS-based UHPLC-MS/MS method was more rapid and convenient than other methods (Table 4). The analysis time of SFN and I3C could be achieved in 8 min without a complicated gradient procedure. Additionally, this is the first time that QuEChERS was applied to extraction of SFN and I3C from vegetables, avoiding the tedious extraction step, which completely eliminates matrix effects during the MS detection with good sensitivity and excellent efficiency.

## 3. Materials and Methods 

### 3.1. Chemicals and Materials

Sulforaphane and indole-3-carbinol standards were obtained from Shanghai Aladdin Technology Co., Ltd. (Shanghai, China). Methanol, *n*-hexane, methyl tert-butyl ether (MTBE), ethyl acetate (EtOAc), dichloromethane (DCM) and ammonium formate of HPLC grade were purchased from Shanghai Aladdin Technology Co., Ltd. (Shanghai, China). C18, N-propyl ethylenediamine (PSA), graphitized carbon black (GCB) was purchased from Agilent Technologies Co., Ltd. (Beijing, China). Anhydrous magnesium sulfate and sodium chloride were purchased from Sinopharm Chemical Reagent Co., Ltd. (Beijing, China). All other chemicals and organic solvents were of analytical reagent grade. Ultra-pure water (18 mΩ) was obtained from a Milli-Q water purification system from Millipore Co., Ltd. (Milford, CT, USA). Rapeseed was obtained from the Oil Crop Research Institute, Chinese Academy of Agricultural Sciences (Wuhan, China). 

Sulforaphane and indole-3-carbinol standard (10.0 mg (accuracy to 0.01 mg)) were dissolved with 10 mL of methanol, for a stock solution of 1.0 mg/mL. A series of standard solutions were diluted with methanol to appropriate concentrations for the calibration curves, and all the standard solutions were stored at 4 °C in darkness.

### 3.2. Sample Preparation

The leaves and stems from rapeseed (*N* = 50) samples were crushed, pounded and processed separately. Each sample 0.50 g (accurate to 0.01 g) was placed in a 50 mL centrifuge tube and vortexed for 1 min with 10 mL of water, and immediately incubated at 45 °C for 2 h. Sodium chloride (200 mg) was added to the sample, then 5 mL × 2 of dichloromethane was vortexed for 1 min and ultrasound-assisted extraction was performed for 10 min. We then added 25 mg of C18 and 80 mg anhydrous magnesium sulfate to a 50 mL centrifuge tube, and then 5 mL of the extracted supernatants were combined with vortex for 5 min. After centrifugation at 4500 rpm for 5 min, the supernatant was transferred and dried using nitrogen stream. Finally, the residual was reconstituted using 1 mL of methanol, and filtered with 0.22 μm organic microporous membrane for UHPLC-MS/MS analysis.

### 3.3. UHPLC-MS/MS Analysis 

#### 3.3.1. Liquid Chromatography

The UHPLC-MS/MS system consisted of Shimadzu UHPLC-30AD and MS-8060 mass spectrometer (Kyoto, Japan). Selective quantification of compounds was performed using the multiple reaction monitoring (MRM) mode. Two compounds were separated by UHPLC using a solvent system consisting of 10 mM ammonium formate-methanol (solvent A; *v*/*v*) and water (solvent B) along with an ACQUITY UHPLC ^®^ BEH C18 (100 mm × 2.1 mm, 1.7 µm) column. The gradient elution program for UHPLC-MS/MS analysis was as follows: 0 min, 40% A; 2.5 min, 45% A; 3 min, 50% A; 3.5 min, 95% A; 4 min, 45% A; 8 min, 40% A. The injection volume was 1 µL, and the flow rate was 200 µL/min. The column temperature was held at 40 °C.

#### 3.3.2. MS Analysis

**Figure 6:**
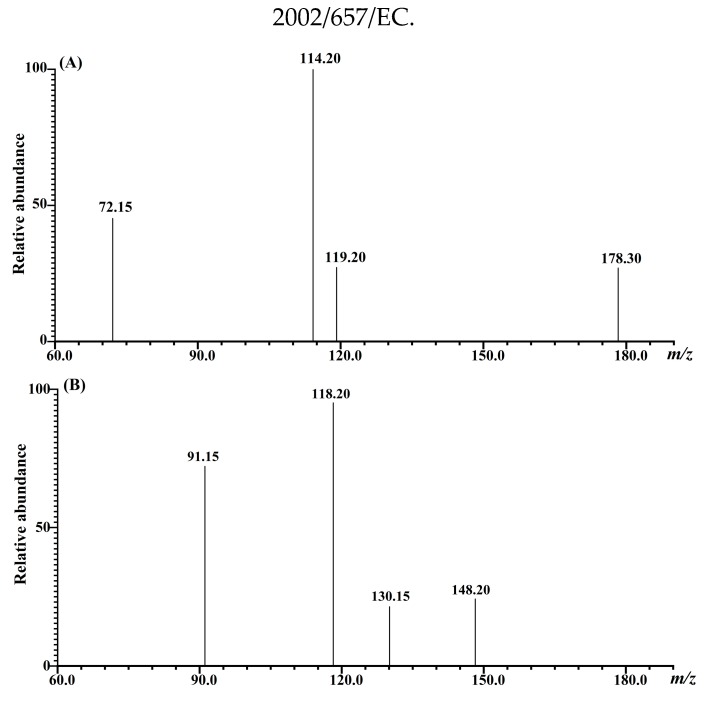
The product ions of sulforaphane and indole-3-carbinol obtained by UHPLC-MS/MS. (**A**) product ions of *m*/*z* 178.3 from sulforaphane standard; (**B**) product ions of *m*/*z* 148.2 from the indole-3-carbinol standard.

Sulforaphane and indole-3-carbinol were optimized using 1 mg/L standard solution on a Shimadzu MS-8060 triple quadrupole tandem mass spectrometer. The collision energy (CE) and the appropriate atomization gas flow rate and drying gas flow rate were optimized. The ion source used an electrospray ion source (ESI), with a temperature of 300 °C. The ion transfer capillary temperature was 275 °C, the spray gas flow rate was 2.5 L/min, the dry gas flow rate was 10 L/min, and the heated gas flow rate was 10 L/min. The heating block temperature was 400 °C, the DL temperature was 250 °C and the scanning was performed in the positive ion mode. The MS/MS parameters for compound analysis were shown in Table 5. The MRM chromatogram of SFN and I3C were identified by comparing the retention time, as well as parent and product ions with the standard solutions. As shown in Figure 6, the most abundant product ions were selected as identification points (IPs), and the other product ions were utilized for qualitative detection.

### 3.4. Statistical Analysis

Results were expressed as average values with three replicates. Data acquisition and processing were performed using the labSolutions software (Kyoto, Japan). Statistical analyses were performed using the @Risk 5.5.1 software package from Palisade Co. (Palisade, Australia). Significant differences were determined by the Student t-test at a significance level of 0.05 (*p* < 0.05).

## 4. Conclusions

In the present study, the QuEChERS-based UHPLC-MS/MS method was developed for the simultaneous determination of sulforaphane and indole-3-carbinol in rapeseed tissues, which achieves simplicity of the extraction and purification procedure and reduces the entire analysis time. The recoveries for sulforaphane and indole-3-carbinol were 96.4% and 97.3%, respectively. The LOD and LOQ for sulforaphane were 0.05 μg/kg and 0.15 μg/kg, and for indole-3-carbinol were 5 μg/kg and 15 μg/kg, respectively. The amount of sulforaphane and indole-3-carbinol in the leaves were higher compared to the stems, which could be attributed to the part, variety, geography, climate and environment conditions. The developed method is valuable for monitoring levels of sulforaphane and indole-3-carbinol in vegetable samples. This analytic method is valuable for studying the nutritional active ingredients of rapeseeds and the breeding of high-quality rapeseed varieties.

## Figures and Tables

**Table 1:** Linear equations, correlation coefficient (R^2^), LODs, LOQs for sulforaphane and indole-3-carbinol.

Compound	Linear Range (μg/kg)	Regression Equation	Correlation Coefficient	LOD (μg/kg)	LOQ (μg/kg)
Sulforaphane	0.1–800	Y = 458,317X − 133,901	0.9999	0.05	0.15
Indole-3-carbinol	15–1000	Y = 42.3285X + 397.599	0.9986	5	15

**Table 2:** Recovery rate, intra-day and inter-day precision for sulforaphane and indole-3-carbinol.

Compound	Spiked Concentration (µg/kg)	Recovery (%)	Intra-Day Precision (*N* = 3, %)	Inter-Day Precision (*N* = 5, %)
Sulforaphane	1/5/25	76.5/91.3/96.4	4.9/5.9/4.1	9.4/9.0/11.3
Indole-3-carbinol	20/50/100	80.2/92.3/97.3	10.3/8.9/7.7	9.9/10.6/9.8

**Table 3:** Quantity of the detected sulforaphane and indole-3-carbinol in rapeseed samples ^a^ (µg/kg).

Rapeseed Tissue (*N* = 50)	Sulforaphane			Indole-3-Carbinol		
	Max	Min	Mean	Max	Min	Mean
Stems	415.3 ± 4.7	ND ^b^	68.5 ± 15.9	131.3 ± 11.8	ND	41.9 ± 12.4
Leaves	1621.8 ± 28.1	14.6 ± 6.7	287.3 ± 24.8 *	879.5 ± 27.9	36.4 ± 5.9	285.4 ± 18.7 *

^a^ Values represent the mean of the triplicate analyses ± the standard deviation. ^b^ ND, below the limit of detection. “*” with brackets represent significant differences of SFN and I3C between the stems and leaves, *p* < 0.05.

**Table 4:** Comparison of the extraction step and LOD with the previous methods.

Matrix	Analytes	Extraction Step	Determination Technique	Analyzed Time (min)	Linear Range	LOD (µg/kg)	Ref.
Broccoli	SFN	LLE	UHPLC–HR MS	20	-	770	[20]
	I3C					420	
Broccoli	SFN	SPE	HPLC-UV	20	5–100	20	[21]
Brassicaceae	SFN	DLLME	LC-DAD	30	-	100	[22]
	I3C					500	
Broccoli	SFN	LLE	UHPLC-MS/MS	3	1.8–897.1	0.53	[24]
Chinese cabbage, mustard	I3C	LLE	HPLC-DAD	65	15–1000	5	[34]
Rapeseed	SFN	QuEChERS	UHPLC-MS/MS	8	0.1–800	0.05	This work
	I3C				15–1000	5	

**Table 5:** Mass scan parameters and retention time for sulforaphane and indole-3-carbinol.

Compound	R.T (min)	Quantitative Ion Pair	CE (eV)	Qualitative Ion pairs	CE (eV)	I.P. ^a^
Sulforaphane	3.014	178.3 > 114.2	19	178.3 > 72.1	20	4
Indole-3-carbinol	2.017	148.2 > 118.2	15	148.2 > 91.1	30	4

^a^ Identification points (IPs) are followed Commission Decision 2002/657/EC.
